# Imaging the impact of sex and age on OATP function in humans: Consequences for whole-body pharmacokinetics and liver exposure

**DOI:** 10.1016/j.apsb.2025.03.030

**Published:** 2025-03-17

**Authors:** Solène Marie, Anne-Lise Lecoq, Louise Breuil, Fabien Caillé, Vincent Lebon, Claude Comtat, Sébastien Goutal, Laurent Becquemont, Michel Bottlaender, Céline Verstuyft, Nicolas Tournier

**Affiliations:** a Université Paris-Saclay, CEA, Inserm, CNRS, BioMaps, Service Hospitalier Frédéric Joliot, Orsay 91401, France; b Département de Pharmacie Clinique, Faculté de Pharmacie, Université Paris-Saclay, Orsay 91400, France; c AP-HP. Université Paris-Saclay, Hôpital Bicêtre, Pharmacie Clinique, Le Kremlin Bicêtre 94270, France; d AP-HP. Université Paris-Saclay, Hôpital Bicêtre, Centre de Recherche Clinique, Le Kremlin Bicêtre 94270, France; e CESP, MOODS Team, INSERM UMR 1018, Faculté de Médecine, Univ Paris-Saclay, Le Kremlin Bicêtre F-94275, France; f AP-HP. Université Paris-Saclay, Hôpital Bicêtre, Service de génétique Moléculaire, Pharmacogénétique et Hormonologie, Le Kremlin Bicêtre 94270, France

**Keywords:** Liver exposure, Imaging, Drug transporter, Drug–drug interaction, Drug-induced liver injury, Pharmacokinetics, Solute carrier, Gender

## Abstract

Organic anion-transporting polypeptides (OATP) transporter function, which mediates many drugs' liver uptake, was investigated as a molecular determinant of pharmacokinetic variability. Whole-body PET imaging using ^11^C-glyburide, a metabolically stable OATP probe, was performed in 16 healthy humans. Ten subjects underwent another ^11^C-glyburide PET acquisition after OATP inhibition using rifampicin. Subjects were sorted according to age and sex: males<30y (24.0 ± 3.2 y, *n* = 7), males>50y (57.5 ± 5.6 y, *n* = 4), and females>50y (60.6 ± 2.4 y, *n* = 5). The blood-to-liver transfer rate (*k*_uptake_) was estimated to describe OATP function. Rifampicin decreased *k*_uptake_ (−73 ± 13%, *P* < 0.001) and liver exposure (−50 ± 10%, *P* < 0.001) while increasing exposure in blood (+24 ± 24%, *P* < 0.01), myocardium, spleen, and brain (*P* < 0.05). No evidence of extra-hepatic rifampicin-inhibitable transport of ^11^C-glyburide was found. Baseline liver exposure was 42.6 ± 18.4% higher (*P* < 0.05) in females>50y compared with males>50 y, consistent with higher *k*_uptake_ values (*P* < 0.05), with negligible impact on blood exposure (*P* < 0.05). In males, neither liver exposure, blood exposure, nor *k*_uptake_ were affected by aging (*P* < 0.05). *k*_uptake_ was positively and negatively correlated with liver (*P* < 0.01, *R*^2^ = 0.78) and blood (*P* < 0.01, *R*^2^ = 0.40) exposures respectively. The impact of OATP function (*k*_uptake_) on liver exposure was 4-fold more pronounced than on blood exposure. OATP function may thus drive important sex-related differences in liver exposure, which were not discernible through conventional blood-based pharmacokinetics.

## Graphical abstract

Organic anion-transporting polypeptides (OATP) function, which mediates the uptake transport of many drugs by hepatocytes, is higher in females, resulting in greater liver exposure. This sex difference was not discernible through blood-based pharmacokinetics.

**Figure:**
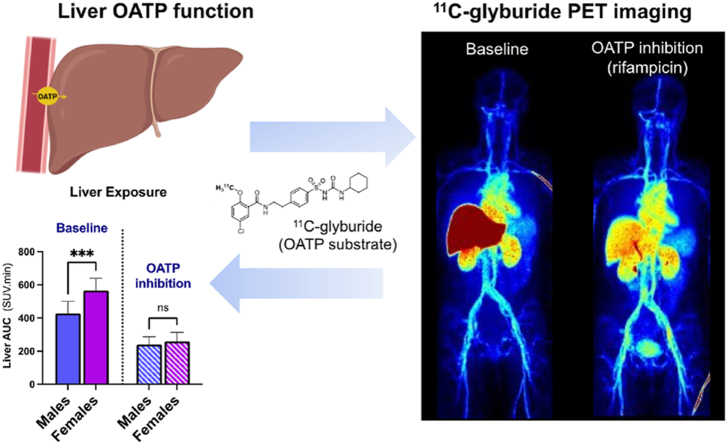

## 1 Introduction

Population characteristics such as sex and aging may be associated with clinically significant differences in pharmacokinetics (PK), with consequences for drug efficacy and safety^1^. Such pharmacokinetic variability is often observed at the latest stages of drug development and even once the investigated drug is on the market. Anticipating pharmacokinetic variability may help optimize dose regimens to improve drug efficacy and tolerance in the targeted populations. However, in most situations, the molecular determinants leading to PK variability associated with sex or aging remain largely unknown^2^^,^^3^. In this context, sex- or aging-related differences in the function of most known drug transporters remain to be clarified in humans^4^^,^^5^.

Transporters of the organic anion-transporting polypeptides family (OATP), also known as Solute Carrier (SLCO) transporters, are considered important for PK^6^^,^^7^. In particular, OATPs expressed by hepatocytes (OATP1B1, OATP1B3, and OATP2B1) are known to play a key role in the hepatobiliary elimination of many drugs by mediating their uptake by hepatocytes^8^^,^^9^. Inhibition of liver OATPs is associated with decreased hepatobiliary clearance and may lead to clinically significant drug–drug interactions (DDIs) between OATP substrates and inhibitors^6^. Studies have also demonstrated a clinically significant decrease in the clearance of OATP substrates such as statins, repaglinide, atrasentan, irinotecan, and many others^6^^,^^10^. Polymorphisms of *SLCO* genes, especially OATP1B1, were associated with this decrease; these results hold potentially significant consequences for toxicity^11^. Some studies have focused on potential changes in OATP expression associated with sex and aging in liver samples from animals or humans^5^^,^^12^, ^13^, ^14^. It may nonetheless be hypothesized that changes in OATP function may account for the observed sex- or age-related PK variability in humans.

Clinical pharmacokinetic studies indirectly estimate the impact of changes in OATP function through changes in the plasma kinetics of the substrate drugs ^4^^,^^15^. However, this approach cannot accurately predict the extent of changes in OATP function at the blood-liver level. Additionally, from a drug efficacy or toxicity perspective, the target tissue concentration rather than plasma concentration is the most relevant. Unfortunately, changes in plasma exposure cannot accurately predict changes in exposure of target/vulnerable organs ^16^. This unknown particularly holds for liver exposure which may lead to drug-induced liver injury (DILI) ^17^.

Hepatic OATP function can be non-invasively and quantitatively studied through ^11^C-glyburide, a recently developed positron emission tomography (PET) probe^18^^,^^19^. ^11^C-glyburide is predominantly transported by OATP1B1, which mediates its uptake from the blood to the liver^20^^,^^21^. The clinical validation of ^11^C-glyburide as a PET probe for OATP, conducted in healthy young males, showed its specificity and suitable sensitivity to detect changes in liver transport function ^18^.

This study conducted whole-body dynamic (WB-4D) PET-MR (magnetic resonance) experiments using ^11^C-glyburide to explore differences in OATP function associated with sex and aging. Liver OATP function and WBPK of ^11^C-glyburide were compared in healthy males and females (>50y). Data obtained in males >50y were compared to those obtained in younger males (<30y) using the same method. All subjects were assessed for single nucleotide variants (SNVs) of *SLCO* genes to consider any difference in genetic polymorphism.^10^

## 2 Material and methods

### 2.1 Subjects and study design

The study protocol was approved by an Ethic Committee (CPP IDF5: 17041, Study registration EudraCT 2017-001703-69). Experiments were conducted with respect to the 1975 Declaration of Helsinki. All subjects had a first medical examination where the inclusion criteria were checked by the clinical investigator. A written informed consent was signed after the subjects received all information about the study. A blood sample was collected for pharmacogenetic analysis, to genotype the *SLCO* genes encoding for OATP transporters.

Twenty-four volunteers were included in the study. Eight of them could not participate in imaging sessions due to technical issues. The subjects were classified into three groups: males below 30 years old (M < 30 y, 24.0 ± 3.2 y, *n* = 7), males above 50 years old (M > 50 y, 57.5 ± 5.6 y, *n* = 4), and post-menopausal females above 50 years old (F > 50 y, 60.6 ± 2.4 y, *n* = 5), Details about the groups and the subjects are provided in Table 1. Previously reported validation of ^11^C-glyburide for OATP imaging was performed with the data of the males <30y group ^18^.

**Table 1:** Characteristics of healthy volunteers undergoing ^11^C-glyburide PET imaging. The wild-type genotypes are AA for *SLCO1A2*, TT for *SLCO1B1*, GG for *SLCO1B3*, GG for *SLCO2B1* ex5, and CC for *SLCO2B1* ex10. Homozygous allelic variants are highlighted in grey.

Group	Volunteer number	Age (y)	Weight (kg)	Rifampicin scan	Genotype				
					*SLCO1A2* c.516A > C (rs11568563)	*SLCO1B1* c.521T > C (rs4149015)	*SLCO1B3* c.699G > A (rs7311358)	*SLCO2B1* ex5 c.601G > A (rs35199625)	*SLCO2B1* ex10 c.1457C > T (rs2306168)
M < 30 y	1	30.2	59	No	AA	TT	AA	GG	CC
	2	24.7	73	Yes	AC	TT	AA	GG	CC
	3	24.5	66	Yes	AA	CT	AA	GG	CC
	4	22.2	81	Yes	AA	TT	GA	GG	TT
	5	20.3	83	Yes	AC	TT	AA	GG	CC
	6	21.4	61	No	AA	CT	GA	GG	CC
	7	24.7	87	Yes	AA	TT	AA	GG	CC
M > 50 y	8	59.3	81	No	AA	TT	AA	GG	TC
	9	63.5	88	Yes	AA	TT	AA	GG	CC
	10	57.8	74	No	AA	TT	GG	GG	CC
	11	50.2	81	No	AA	CT	AA	GG	CC
F > 50 y	12	59.6	66	Yes	AA	CC	GA	GG	CC
	13	58.8	72	Yes	AA	CT	AA	GG	CC
	14	58.8	67	Yes	AA	CT	AA	GG	CC
	15	61.2	61	Yes	AA	CT	GA	GG	CC
	16	64.6	53	No	AA	TT	AA	GG	CC

Imaging sessions were held on a single day. Baseline WB-4D PET acquisition started with intravenous (i.v.) bolus of ^11^C-glyburide. When possible, at least 3 h later after the first acquisition, a second ^11^C-glyburide WB-4D PET scan was performed after infusion of rifampicin, used as an inhibitor of OATP, as previously described ^18^ (9 mg/kg diluted in glucose 5% perfused, within 45 min of ^11^C-glyburide injection, Rifadin®, Sanofi-Aventis, Gentilly, France). Sixteen volunteers underwent a baseline ^11^C-glyburide WB-4D PET acquisition. Ten of them underwent a second WB-4D PET in the presence of OATP inhibition (*n* = 5, 1, and 4, respectively for M < 30 y, M > 50 y, and F > 50y).

### 2.2 Candidate genetic polymorphisms analysis

Methods for genotyping *SLCO1A2* c.516A > C (rs11568563), *SLCO1B1* c.521T > C (rs4149015), *SLCO1B3∗4* c.699G > A (rs7311358), *SLCO2B1* ex5 c.601G > A (rs35199625), and *SLCO2B1* ex10 c.1457C > T (rs2306168) are reported as supporting information.

### 2.3 PET acquisition

Pharmaceutical grade production of ^11^C-glyburide, obtained using a previously reported method ^22^, is described as supporting information. WB-4D PET acquisitions were performed using a SIGNA® PET/MR scanner (GE Healthcare, Waukesha, WI, USA). The subjects were positioned on the patient bed, and MR localization acquisitions were performed. One venous catheter was inserted in each arm to inject ^11^C-glyburide (1 min bolus) and blood sampling, respectively. Blood sampling was performed to i*)* determine the binding to plasma proteins before ^11^C-glyburide injection (*T* = 0) ^57^ and ii*)* assess the unchanged fraction of ^11^C-glyburide in plasma (*T* = 0, 5, 10, 15, and 30 min after ^11^C-glyburide injection, supporting information). The mean injected dose of ^11^C-glyburide for baseline scans was 167 ± 56 MBq and 174 ± 53 MBq for the second scans. The mean specific radioactivity at the time of injection was 9.5 GBq/μmol and the corresponding mass of glyburide injected was 12.5 ± 6.2 μg. PET acquisition started with the injection of ^11^C-glyburide. The first 3-min dynamic mono-bed acquisition was positioned on the abdomen (16 frames of 10 s), to capture uptake in the liver and some abdominal organs. This acquisition was immediately followed by repeated multi-bed whole-body acquisitions performed over the next 30 min (11 5-bed position acquisitions of 2.5 min, over a whole-body axial field-of-view of 25 cm).

### 2.4 PET data analysis

PET images were reconstructed using a 3D-iterative reconstruction algorithm, including resolution modeling and time-of-flight. Data was corrected for radioactive decay, attenuation, and random and scattered coincidences. Attenuation correction was based on a 2-point Dixon MR sequence (MRAC). Volumes of interest (VOIs) were delineated for tissues (liver, kidneys, spleen, myocardium, pancreas, brain, and muscle) from PET and/or co-registered MRAC images using the PMOD® software (version 3.9, PMOD Technologies LLC, Zurich, Switzerland). An additional VOI was delineated which includes the left ventricle and the aorta to obtain an image-derived input function (IDIF). Validation of this method, performed in subjects of the males <30 y group, has already been reported and demonstrated a good correlation between IDIF and data from arterial blood sampling^18^. Urinary bladder was not investigated since our preliminary study using ^11^C-glyburide demonstrated that radioactivity detected in urine almost exclusively corresponded to radiometabolites ^18^.

Time–activity curves (TACs) were generated by calculating the mean radioactivity in these VOIs (kBq/cc). The TACs were expressed in standardized uptake values (SUV, unitless), generated by correcting the radioactivity in the VOIs for injected dose (kBq) and body weight (g). Areas under the TACs (AUC, expressed in SUV.min) were calculated from 0 to 30 min for each TAC to describe tissue exposure. Ratios between AUC in each tissue and arterial AUC estimated by IDIF (*K*_p_ = AUC_tissue_/AUC_blood_) were also calculated from 0 to 30 min to estimate tissue distribution.

The transfer constant (*k*_uptake_) of ^11^C-glyburide from blood to the liver was estimated using the integration plot analysis ^23^^,^^24^ from 5 to 30 min after ^11^C-glyburide injection using Eq. (1):

(1) $$\frac{C_{\text{liver},t}}{C_{\text{blood},t}}=k_{\text{uptake}}\times \left(\frac{{\text{AUC}}_{\text{blood},0-t}}{C_{\text{blood},t}}\right)+V_{E}$$

where *C*_liver,*t*_ and *C*_blood,*t*_ represent the concentrations of ^11^C-glyburide in the liver and blood (IDIF) respectively at time *t*. AUC_blood,0–*t*_ represents the area under the IDIF TAC from time 0 to *t*. *k*_uptake_ corresponds to the slope of the linear regression plot. *V*_E_ represents the initial distribution volume in the liver at time 0.

### 2.5 Data and statistical analysis

Data is reported as the mean ± SD (standard deviation). Differences between multiple groups were analyzed by a mixed-effect analysis followed by Tukey’s multiple-comparison test. The level of statistical significance was set to a *P*-value of less than 0.05. Linear regression and statistical comparisons were performed using GraphPad Prism® software (version 10.0, San Diego, CA, USA).

## 3 Results

### 3.1 Subjects and SLCO genotypes

Results of the genotyping of the genes encoding for OATP transporters are presented in Table 1. All subjects were wild-type carriers for the *SLCO2B1* ex5 (rs35199625) gene. Allelic variants were identified for all the other tested genes, with a range from one carrier of heterozygous mutation for *SLCO2B1* ex10 (rs2306168) to 6 mutated alleles for *SLCO1B1* (rs4149015). One subject carrier of the homozygous allelic variant was observed for the *SLCO2B1* ex10 (rs2306168, M < 30 y) and *SLCO1B1* (rs4149015, F > 50 y) genetic polymorphisms. Regarding the *SLCO1B3* (rs7311358, M > 50 y) polymorphism, 11 subjects were carriers of the c.699G > A mutation, which is predominant in Europe^25^.

### 3.2 Metabolism and binding to plasma protein

^11^C-glyburide was barely metabolized in both baseline and rifampicin conditions (Supporting Information Fig. S1). In all subjects, regardless of the presence or the absence of rifampicin, parent (unmetabolized) ^11^C-glyburide accounted for 90% or more of the plasma radioactivity during PET acquisition in the three groups of subjects. It was therefore decided not to correct blood data from radiometabolites given their limited impact on PET quantification. ^11^C-glyburide was highly bound to plasma proteins with a mean of 99.7 ± 0.6% in baseline condition and 99.3 ± 1.1% in rifampicin condition (Supporting Information Table S1), implying negligible impact of rifampicin on the free fraction of ^11^C-glyburide in plasma. Blood kinetics could therefore be described by the IDIF for kinetic modeling, as previously validated ^18^.

### 3.3 Liver and blood data

**Figure 1:**
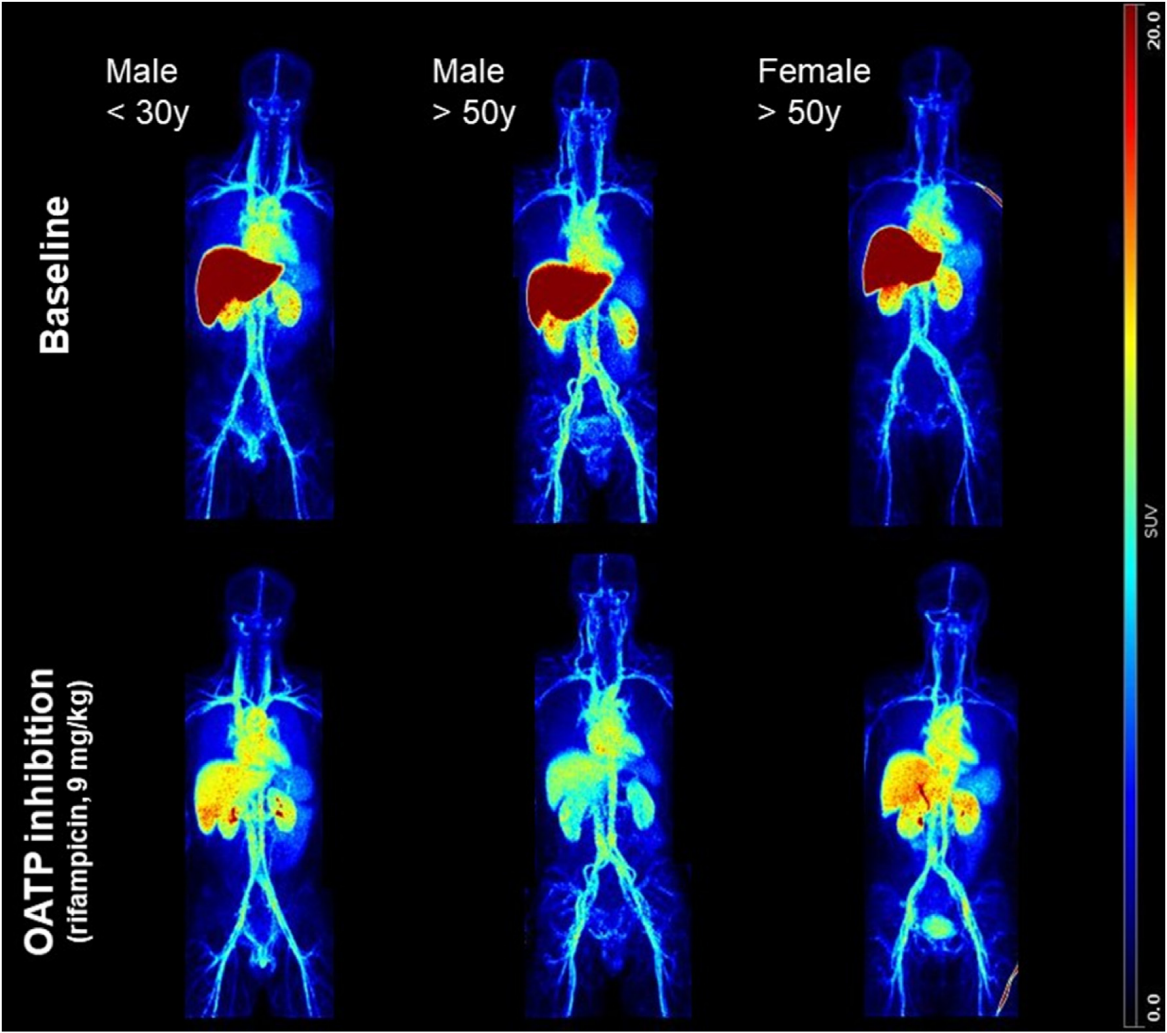
Whole-body PET images of ^11^C-glyburide distribution in baseline condition and after treatment with rifampicin (9 mg/kg i.v.) according to age and sex. Images are the maximum intensity projections of the frames summed from 0 to 30 min and are expressed in standardized uptake values (SUV).

Representative PET images of the whole-body distribution obtained in each group display the predominant uptake of ^11^C-glyburide by the liver (Fig. 1). Liver uptake was visually reduced by OATP inhibition. Radioactivity in the circulation was obvious, probably reflecting the binding of ^11^C-glyburide to plasma proteins.

**Figure 2:**
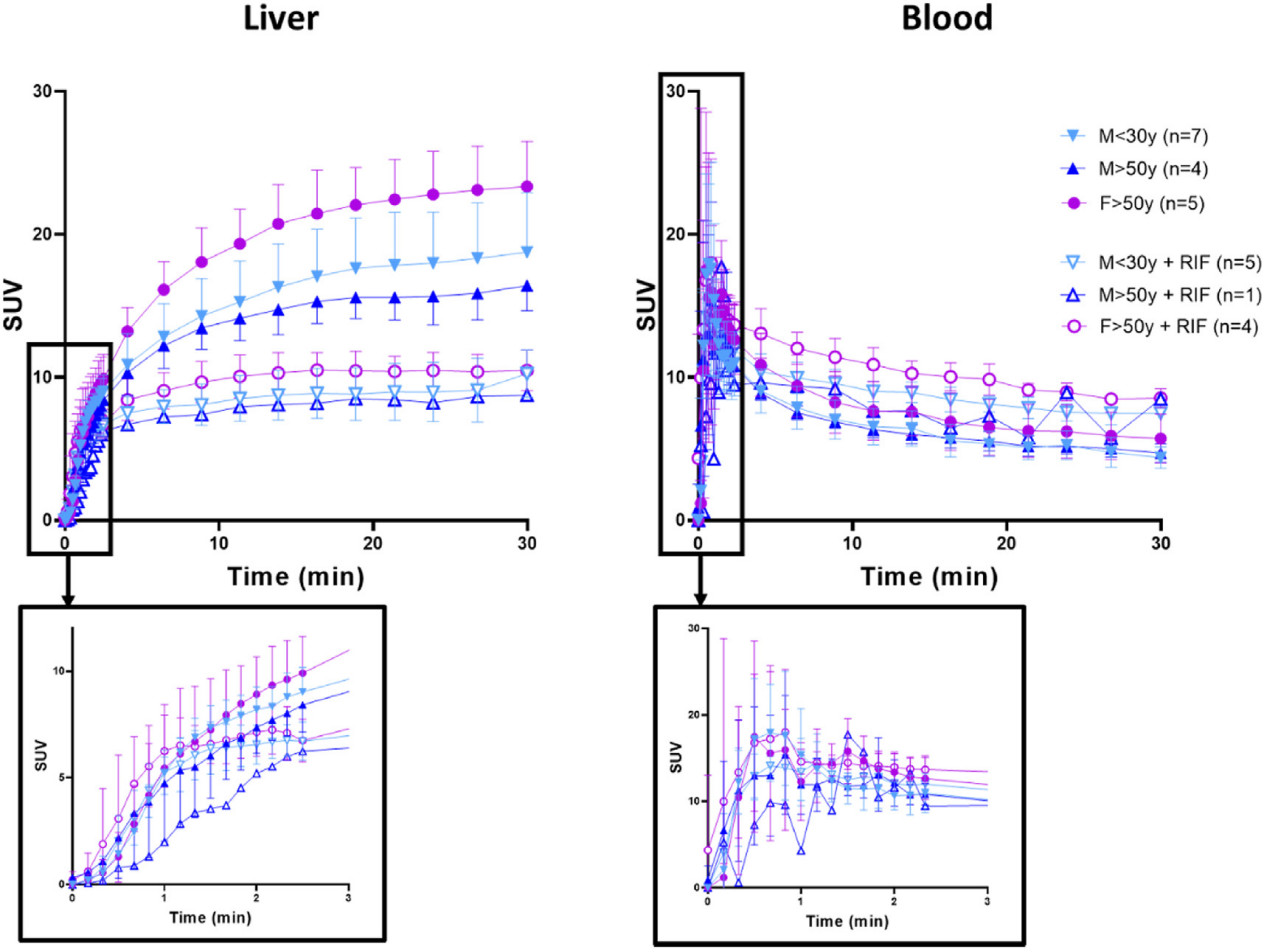
Time‒activity curves of ^11^C-glyburide in liver and blood (image-derived) in the baseline condition and after treatment with rifampicin (RIF, 9 mg/kg i.v.). Data is mean ± SD.

**Figure 3:**
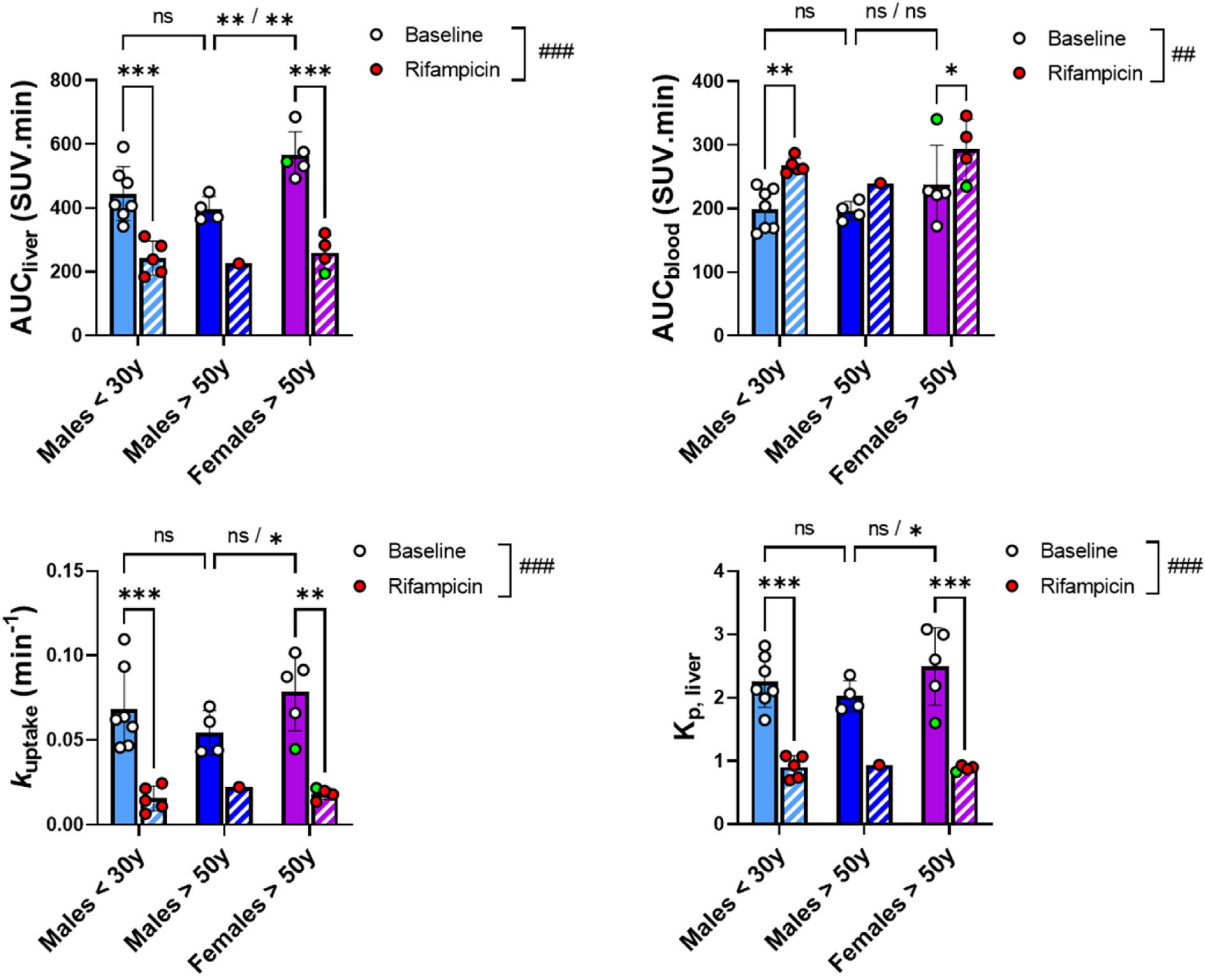
Areas under the time activity curves (AUC, 0–30min) in liver and blood, transfer constants (*k*_uptake_) from blood to liver, and K_p,liver_ (AUC_liver_/AUC_blood_) of ^11^C-glyburide. *k*_uptake_ was assessed from integration plot analysis. White signs (and full bars) are used for baseline condition and red signs (and hatched bars) after treatment with rifampicin. The green signs indicate the subject with the homozygous mutation of the *SLCO1B1* gene. Data is mean ± SD. Statistical comparisons were performed using a mixed-effect analysis. The significance of the effect of rifampicin on the whole population is reported as ^*##*^*P* < 0.01 and ^*###*^*P* < 0.001. The Tukey’s multiple-comparison test was performed with ∗*P* < 0.05, ∗∗*P* < 0.01, ∗∗∗*P* < 0.001, and ns non-significant. ∗∗/∗∗, ns/∗, ns/ns show the outcome of comparison without or with the exclusion of the green sign subject.

The TACs presented in Fig. 2 confirm the high uptake of ^11^C-glyburide by the liver. In baseline conditions, SUV_max_ liver was 18.8 ± 4.2 for M < 30 y, 16.4 ± 1.8 for M > 50 y, and 23.3 ± 3.2 for F > 50 y. AUC_liver_ was similar in M > 50y compared to M < 30 y (−10.7 ± 8.8%, *P* > 0.05), suggesting a limited impact of aging on liver exposure (Fig. 3). However, liver exposure was significantly higher in F > 50y compared with age-matched males (M > 50 y, +42.6 ± 18.4%, Fig. 3). Changes in liver exposure did not translate into significant changes in blood exposure neither between M > 50 y *versus* F > 50 y, nor between M < 30 y and M > 50 y (Figure 2, Figure 3). Significantly, higher liver exposure in females was also found when compared with pooled male data (M < 30 y + M > 50 y, *n* = 11, *P* < 0.001, Supporting Information Fig. S2). Only one subject belonging to the F > 50 y group presented the homozygous mutation on the *SLCO1B1* gene (Table 1). She differed from the rest of the group in terms of blood exposure (AUC_blood_ = 340 SUV.min *vs* 212 ± 26 SUV.min for the rest of the group, Fig. 3, Table S1) but not for the liver exposure (AUC_liver_ = 544 SUV.min *vs* 572 ± 83 SUV.min for the rest of the group, Fig. 3, Table S1). Such behavior was not observed for subjects with either homozygous mutations of the *SLCO1B3* (M > 50 y group), *SLCO2B1* (M < 30 y group) genes, or heterozygous mutations of the investigated genes.

OATP inhibition using rifampicin drastically decreased liver exposure of ^11^C-glyburide in all studied groups (−50 ± 10%, *P* < 0.001, Fig. 3, Table S1), which translated into a significant increase in blood exposure (+24 ± 24%, *P* < 0.01, Fig. 3, Table S1). This trend was similarly observed across study groups. In the presence of rifampicin, levels of liver exposure were not different across study groups (*P* > 0.05, Fig. S2).

The *k*_uptake_, which describes the blood-to-liver transfer of ^11^C-glyburide, was significantly decreased by rifampicin (Fig. 3). The global magnitude of decrease in *k*_uptake_ was −73 ± 13% (*P* < 0.001, *n* = 10). Low and similar *k*_uptake_ values were obtained in the presence of rifampicin, with no significant difference between groups (*P* > 0.05, Fig. 3). Mean baseline *k*_uptake_ in F > 50y (*n* = 5) was 45 ± 42% higher than that of M > 50 y, although non-significant when considering the entire group (*n* = 5, Fig. 3). However, statistical significance was reached (+60 ± 28%, *P* < 0.05, *n* = 4) when removing the subject with the homozygous mutation at the *SLCO1B1* gene from the F > 50y group (Fig. 3). This result was confirmed when pooling together the male data (*P* < 0.05, Fig. S2). Importantly, the magnitude of decreased *k*_uptake_ induced by rifampicin was strongly correlated with baseline *k*_uptake_ (*P* < 0.001, *R*^2^ = 0.95, Supporting Information Fig. S3), consistent with the negligible *k*_uptake_ values of ^11^C-glyburide in the presence of OATP inhibition.

**Figure 4:**
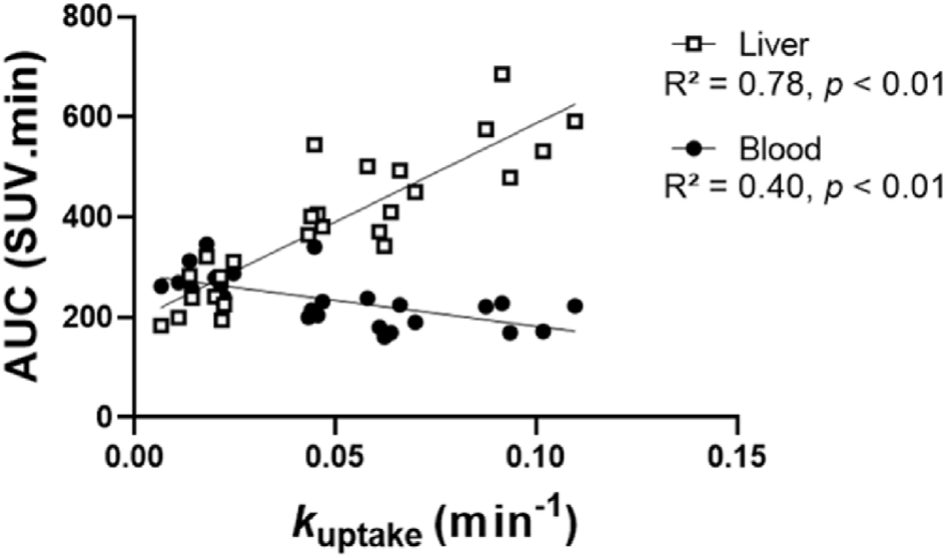
Correlation between *k*_uptake_ and the areas under the curves (AUCs) of ^11^C-glyburide in liver and blood. Linear regression was used to test the correlation between liver or blood exposure and *k*_uptake_.

When considering subjects of all 3 groups, in the presence and the absence of rifampicin, a significant and positive correlation was observed between *k*_uptake_ and the liver exposure (*P* < 0.01, *R*^2^ = 0.78, slope = 3937 SUV.min^2^, Fig. 4). A negative correlation was also observed between *k*_uptake_ and blood exposure (*P* < 0.01, *R*^2^ = 0.40, slope = −1036 SUV.min^2^, Fig. 4). This shows that OATP-mediated uptake governs both liver and blood exposures. However, the impact of OATP-mediated transport on liver exposure was ∼4-fold higher than that on blood exposure.

### 3.4 WBPK of ^11^C-glyburide

**Figure 5:**
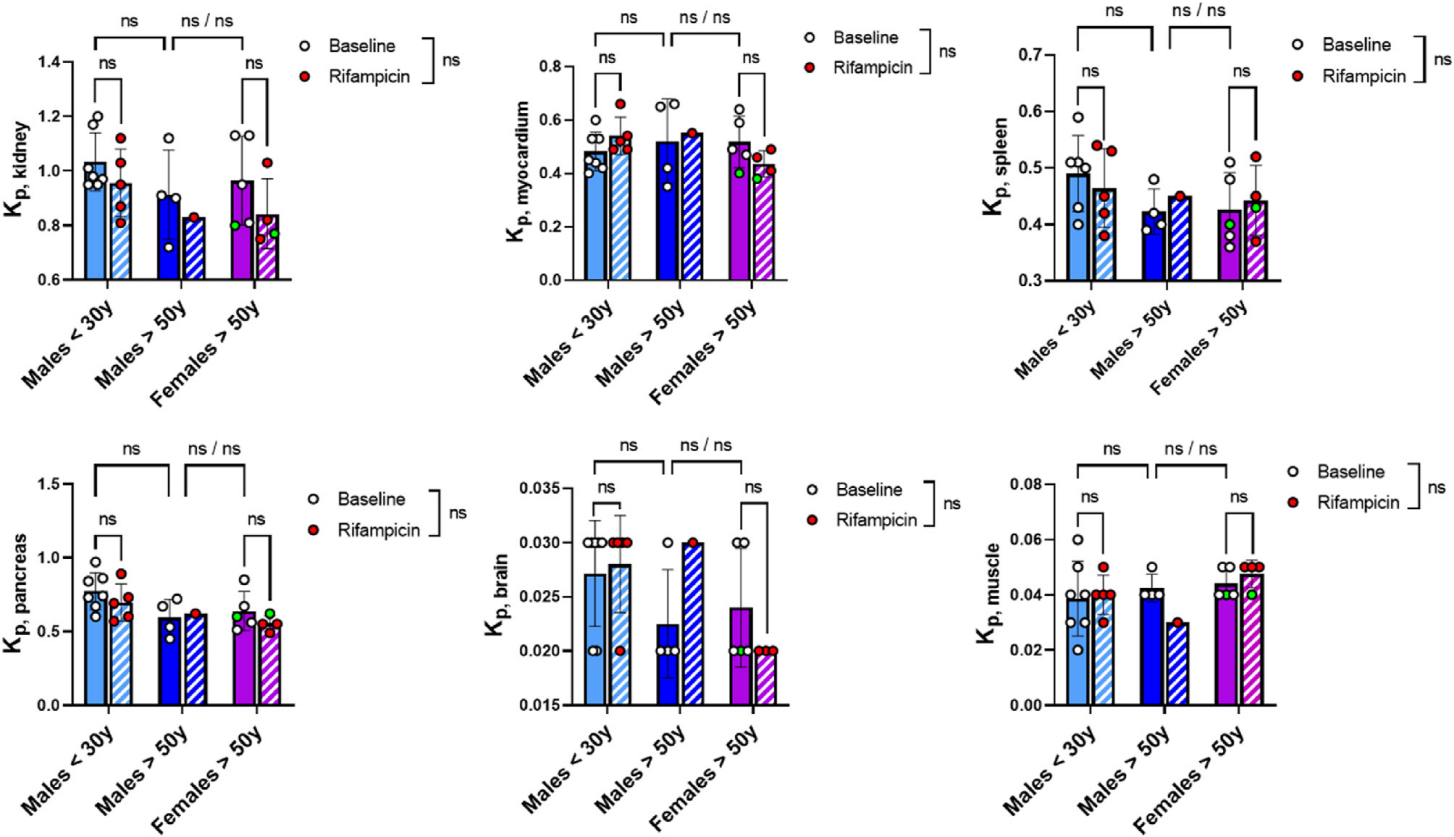
Ratios between AUC in each tissue and blood AUC (*K*_p,tissue_ = AUC_tissue_/AUC_blood_, 0–30min). White signs (and full bars) are used for baseline condition and red signs (and hatched bars) after treatment with rifampicin. The green signs indicate the subject with the homozygous mutation of the *SLCO1B1* gene. Data is mean ± SD. Statistical comparisons were performed using a mixed-effect analysis. The significance of the effect of rifampicin on the whole population is reported as ns non-significant. The Tukey’s multiple-comparison test was performed with ∗*P* < 0.05 and ns non-significant. ns/ns shows the outcome of comparison without or with the exclusion of the green sign subject.

The *k*_uptake_ could not be reliably estimated for most organs except the liver, probably due to low or extremely rapid uptake by these organs. The WBPK of ^11^C-glyburide was therefore described by the tissue/plasma ratio (*K*_p,tissue_ = AUC_tissue_/AUC_blood_)*.* In addition to the liver (*K*_p_ = 2.26 ± 0.23, Fig. 3), substantial uptake of ^11^C-glyburide was observed in the kidneys (*K*_p_ = 0.97 ± 0.06), pancreas (*K*_p_ = 0.67 ± 0.09), myocardium (*K*_p_ = 0.51 ± 0.02), and spleen (*K*_p_ = 0.45 ± 0.04) (Fig. 5).

Consistent with liver *k*_uptake_ values, rifampicin significantly decreased *K*_p,liver_ (Fig. 3, Table S1). However, rifampicin did not impact *K*_p_ in any other organ than the liver, suggesting the absence or limited impact of rifampicin-inhibitable transport (Fig. 5, Table S1). As a consequence of increased blood exposure (AUC_blood_), rifampicin significantly increased tissue exposure (AUC_tissue_) in some organs including the myocardium (*P* < 0.05), spleen (*P* < 0.05) and brain (*P* < 0.001, *n* = 10, Supporting Information Fig. S4).

## 4 Discussion

This study, performed in a cohort of healthy subjects of different sex and age, confirms that ^11^C-glyburide benefits from metabolic stability, in either the presence or the absence of rifampicin^18^. *In vitro* hepatocyte experiments have shown that OATP-mediated uptake (predominantly by OATP1B1) accounts for 98% of the total liver uptake of glyburide, so the estimated proportion of metabolites in the liver is <10% ^26^. Different studies have also reported a higher affinity of glyburide for OATP1B1, with the lowest K_m_ (1.2–2.0 μmol/L), compared to other OATPs (15–17 μmol/L and 6.3 μmol/L for OATP1B3 and OATP2B1 respectively) ^27^. More recently, it was estimated that OATP function accounts for 82% of the total liver uptake of glyburide (45% by OATP1B1, 26% by OATP1B3, and 11% by OATP2B1) using the proteomics-informed relative expression factor approach ^21^. These results suggest that the PET signal predominantly reflects the parent (unmetabolized) compound so that the blood-to-liver transfer (*k*_uptake_) can be reliably estimated for ^11^C-glyburide, which is a major asset compared with previous PET probes^24^^,^^28^^,^^29^. The dramatic decrease in *k*_uptake_ precipitated by the OATP inhibitor rifampicin supports both the sensitivity of ^11^C-glyburide to detect changes in OATP function and its specificity for OATP.

The strong correlation between individual *k*_uptake_ values and corresponding liver or blood exposure suggests that liver OATP function, which occurs at the sinusoidal pole of hepatocytes ^7^, governs both the liver exposure and peripheral kinetics of ^11^C-glyburide as a rate-determining factor. Therefore, our imaging data supports the relevance of the “extended clearance concept,” which assumes that the transmembrane permeation process in elimination organs may predict the overall clearance of drugs ^30^. Notably, the impact of change in *k*_uptake_ on liver exposure was ∼4-fold more pronounced than that on blood exposure. This result illustrates that estimation of OATP function by considering blood concentration of endogenous or exogenous probe substrates only ^4^^,^^31^ may considerably underestimate the impact of these transporters on liver exposure.

Two factors of variability in OATP function were investigated in this study. The most important result is the higher liver exposure observed in females compared with age-matched males after injection of the same dose of ^11^C-glyburide. Higher liver exposure was linked to higher OATP function, as estimated by *k*_uptake_. This outcome points to sex differences in liver exposure to drugs as a consequence of sex-related differences in OATP function. This *in vivo* result is consistent with the 1.3-fold higher expression of the *SLCO1B1* gene in females, measured in human samples ^5^^,^^13^. However, previous proteomic studies based on human liver samples obtained from a large cohort, did not show a significantly higher abundance of OATP1B1 in females compared with males, although considerable variability was observed ^32^^,^^33^. Western-blot analysis has previously revealed a trend toward a higher abundance of liver OATP transporters in females *vs* males. This cohort better matched the population of our study (56 ± 12 y, 7 males, 10 females), however, this trend was not statistically significant^34^.

Our results suggest that sex-biased OATP function, which drives higher liver exposure in females, may account for the higher prevalence of DILI in females^35^^,^^36^. It was shown that females have a 1.5- to 1.7-fold greater risk of developing DILI and the female/male ratio increases after the age of 49 years, suggesting a higher susceptibility to DILI after menopause^37^. Unfortunately, ^11^C-glyburide was not performed in young females for sex comparison in the young population.

Importantly, sex-related differences in OATP function—as well as liver exposure—could not be predicted from blood kinetics of ^11^C-glyburide, even when excluding the subject with the homologous mutation of the *SLCO1B1* gene that shows particularly high baseline blood exposure compared with the rest of the group. Indeed, changes in blood exposure to ^11^C-glyburide drastically underestimated changes in liver exposure. This observation is consistent with PK studies performed in larger cohorts that did not report any change in blood kinetics of glyburide between healthy males and females >60 y^38^. Neither was the plasma kinetics of pravastatin, another OATP1B1 substrate, different between males and females 39, 40, 41.

Aging is the second factor of variability investigated in this study. The impact of normal aging on liver structure and function is well known, with a significant decrease in total liver volume, blood flow, and vascular volume^42^. The impact of age-related decline in renal function on drug clearance is also well established and allows for dose optimization in elderly patients^43^. In contrast, the impact of aging on liver transport and metabolism remains unclear ^43^^,^^44^. Our results do not support any significant impact of aging on liver OATP function, at least in males. Studies comparing the impact of aging on the plasma PK of OATP substrates are often inconclusive due to the frequent involvement of several other elimination pathways^4^. *Ex vivo* studies using proteomic or western-blot analysis reported no correlation between age and the abundance of OATP transporters^32^, ^33^, ^34^. We also found a limited impact of aging on blood or liver exposure to ^11^C-glyburide, which is consistent with previous studies showing no or limited impact of aging on blood exposure to orally administered glyburide in either healthy subjects^38^ or patients with type 2 diabetes^45^. One of these studies reported a slower absorption in older subjects; however, the absorption phase could not be investigated in our study because ^11^C-glyburide was i.v. administered. Moreover, studies reported a negligible impact of aging on the disposition of the OATP1B1 substrate pravastatin in the plasma of healthy subjects so dose adjustment in the older population is not required for this drug^40^^,^^41^.

The liver perfusion of subjects included in this study has not been measured. However, age-related decline in liver perfusion is well established^46^. Our results suggest that OATP function remains unaltered as the main parameter governing the hepatobiliary elimination of ^11^C-glyburide in older males. This finding suggests that OATP-mediated elimination of ^11^C-glyburide does not depend on liver blood flow or perfusion. From a PET imaging perspective, this may suggest that flow-limited passive diffusion of ^11^C-glyburide, which can be estimated in the presence of rifampicin, has limited importance on the estimation of OATP function using this PET probe. Unfortunately, only one M > 50y underwent a second PET scan after complete OATP inhibition. However, the *k*_uptake_ value for this subject was similar to that observed in the F > 50 y and M < 30 y groups.

The use of WB-4D PET acquisition enabled the study of WBPK of ^11^C-glyburide. Imaging data shows a relatively low uptake of ^11^C-glyburide in organs other than the liver. This low signal did not enable accurate quantification of ^11^C-glyburide in some organs of small size, such as testis (data not shown). Importantly, the *K*_p_ of ^11^C-glyburide was not decreased in any investigated organ except the liver. This result suggests a liver-specific transport for this probe, consistent with the prime importance of OATP1B1, a liver-specific isoform, in mediating its transport. As a consequence, ^11^C-glyburide may lack sensitivity and/or specificity to detect extrahepatic OATP function mediated by OATP2B1 or OATP1A2, whose expression has been detected in other organs^9^. OATP inhibition increased the disposition of ^11^C-glyburide in some organs as a result of enhanced blood exposure due to decreased liver uptake. Altogether, this PET data convincingly illustrates the impact of OATP-mediated DDI on WBPK in humans.

Some limitations exist in this study. Compared with traditional PK studies focusing on blood data, the total number of scanned subjects (*n* = 16 baseline scan, 10 rifampicin scan) is relatively small. This number, nonetheless, is higher than in previous PET imaging studies focusing on liver drug transporters ^24^^,^^47^, ^48^, ^49^. Rifampicin scans could not be performed in all the subjects, with only one rifampicin scan in the M > 50y group. Data obtained from 10 subjects belonging to the different groups who underwent both the baseline and post-rifampicin scans was amply sufficient to show the impact of rifampicin on liver function and WBPK. These results validate ^11^C-glyburide PET imaging as a probe for OATP function. For most probes, drug transporter function is estimated by the magnitude of response to complete inhibition which requires two subsequent PET acquisitions^28^^,^^50^. However, the strong correlation between the baseline *k*_uptake_ and rifampicin-precipitated decrease in *k*_uptake_ suggests that a single baseline ^11^C-glyburide PET scan is sufficient to estimate the total transport capacity of OATP, as previously reported ^18^ (Fig. S3). Our sample size (*n* = 4–7 subjects per group) was sufficient to reveal significant sex-related differences of significant pharmacokinetic importance in liver OATP function and exposure.

The impact of aging on OATP function was only investigated in males because females <30 y were not included. Hormonal regulation of OATPs has been reported ^51^^,^^52^. The importance of hormonal changes during the menstrual cycle for pharmacokinetic variability should be considered ^53^. This consideration may complicate the interpretation of ^11^C-glyburide PET data in pre-menopausal females. Moreover, effective contraception, which may also impact OATP function, is required to conduct PET studies in women of childbearing age. For these reasons, only post-menopausal women were included in our study. It should be noted that sex-related differences in OATP function and liver exposure were found in subjects >50 y, who correspond to the most medicated population, with an increased risk of DDI ^54^.

PET studies using dedicated probes may help untangle the pharmacokinetic importance of the genetic polymorphism of *SLCO* genes whose impact on transporter function has been reported^27^^,^^55^. Our study was not designed to address the impact of genetic polymorphism on OATP function using ^11^C-glyburide PET imaging. Only one subject carrier of the well-characterized *SLCO1B1* no-function homozygous variant c.521 T > C (rs4149056) was scanned and differed from the rest of the group (F > 50 y). Other *SLCO1B3* and *SLCO2B1* genetic polymorphisms did not have such an effect in our cohort. However, ^11^C-glyburide may lack the sensitivity to detect changes in OATP1B3 or OATP2B1 function, given its predominant transport by OATP1B1 ^26^. Polymorphisms on the *SLCO1B1* gene have been classified as highly significant for PK by the *International Transporter Consortium* ^56^. Our data suggests that this polymorphism may have dramatic consequences for liver exposure. Further studies using ^11^C-glyburide PET imaging, including a larger number of subjects selected upon *SLCO1B1* gene polymorphism, should be performed to quantitatively address the impact of this SNV on OATP function and the WBPK using ^11^C-glyburide PET imaging. Our results suggest that genetic polymorphism should be investigated separately in males and females.

## 5 Conclusions

WB-4D PET imaging revealed the tissue kinetics of ^11^C-glyburide in humans. OATP inhibition using rifampicin showed the prime importance of hepatocyte OATP function in controlling liver and blood exposure, with consequences for WBPK. Our results suggest no significant decline in OATP function with aging. However, dramatically higher liver exposure was observed in females >50y compared with age-matched males, which was linked with greater OATP function. This sex difference was not discernible through conventional blood-based pharmacokinetics.

## Author contributions

Solène Marie: Writing – review & editing, Writing – original draft, Visualization, Validation, Supervision, Project administration, Methodology, Investigation, Formal analysis, Data curation, Conceptualization. Anne-Lise Lecoq: Writing – review & editing, Validation, Resources, Project administration, Investigation, Data curation. Louise Breuil: Writing – review & editing, Methodology, Formal analysis, Data curation. Fabien Caillé: Writing – review & editing, Resources, Methodology. Vincent Lebon: Writing – review & editing, Resources, Methodology, Investigation, Funding acquisition. Claude Comtat: Writing – review & editing, Visualization, Software, Formal analysis, Data curation. Sébastien Goutal: Writing – review & editing, Visualization, Resources, Formal analysis, Data curation. Laurent Becquemont: Writing – review & editing, Validation, Project administration, Methodology, Investigation, Data curation, Conceptualization. Michel Bottlaender: Writing – review & editing, Visualization, Validation, Methodology, Investigation, Conceptualization. Céline Verstuyft: Writing – review & editing, Supervision, Methodology, Investigation, Formal analysis, Data curation, Conceptualization. Nicolas Tournier: Writing – review & editing, Writing – original draft, Visualization, Validation, Supervision, Resources, Project administration, Methodology, Investigation, Funding acquisition, Formal analysis, Data curation, Conceptualization.

## Conflicts of interest

The authors have no conflicts of interest to declare.

## Appendix A Supporting information

The following is the Supporting Information to this article:
