# Supplementary material for: Imaging the impact of sex and age on OATP function in humans: Consequences for whole-body pharmacokinetics and liver exposure
Source: Acta Pharm Sin B. 2025 Mar 17;15(5):2736–45. doi: 10.1016/j.apsb.2025.03.030 (PMC12144993; doi:10.1016/j.apsb.2025.03.030)
Supplement: Multimedia component 1 [file mmc1.pdf]

Supporting Information for

Original article

Imaging the impact of sex and age on OATP function in humans: consequences for whole-body pharmacokinetics and liver exposure

Solène Marie[a,b,c,*], Anne-Lise Lecoq[d], Louise Breuil[a], Fabien Caillé[a], Vincent Lebon[a], Claude Comtat[a], Sébastien Goutal[a], Laurent Becquemont[d,e], Michel Bottlaender[a], Céline Verstuyft[e,f], Nicolas Tournier[a,*]

[a]*Université Paris-Saclay, CEA, Inserm, CNRS, BioMaps, Service Hospitalier Frédéric Joliot, 91401 Orsay, France*

[b]*Département de Pharmacie Clinique, Faculté de Pharmacie, Université Paris-Saclay, 91400 Orsay, France*

[c]*AP-HP. Université Paris-Saclay, Hôpital Bicêtre, Pharmacie Clinique, 94270 Le Kremlin Bicêtre, France*

[d]*AP-HP. Université Paris-Saclay, Hôpital Bicêtre, Centre de Recherche Clinique, 94270 Le Kremlin Bicêtre, France*

[e]*CESP, MOODS Team, INSERM UMR 1018, Faculté de Médecine, Univ Paris-Saclay, Le Kremlin Bicêtre, F-94275, France*

[f]*AP-HP. Université Paris-Saclay, Hôpital Bicêtre, Service de génétique Moléculaire, Pharmacogénétique et Hormonologie, 94270 Le Kremlin Bicêtre, France*

Received 18 September 2024 ; received in revised form 28 December 2024 ; accepted 15 January 2025

*Corresponding authors.

E-mail addresses: nicolas.tournier1@universite-paris-saclay.fr (Nicolas Tournier), solene.marie@universite-paris-saclay.fr (Solène Marie).

Candidate genetic polymorphisms analysis

Genomic DNA was extracted from circulating leukocytes using the QIAsymphony robot and QIAsymphony DSP DNA Midi Kit (Qiagen, Courtabœuf, France) according to the manufacturer's protocol.

Genotyping for *SLCO1A2* (rs11568563), *SLCO1B1* (rs4149015), *SLCO1B3*4* (rs7311358), and *SLCO2B1* (rs2306168 and rs35199625) SNVs were performed using Taqman® allelic dis-crimination assay on QuantStudio® 7 (QS7) Flex Real-Time PCR System instrument, (Thermo Fischer, Les Ulis, France) using custom probes and primers or commercialized drug-metabolizing enzyme (DME) kits (Applied Biosystems, Villebon sur Yvette, France).

The PCR amplification protocol for TaqMan assays included a denaturation step at 95°C for 10 min, followed by 40 cycles at 92°C for 15 s, 60°C for 1 min, and 72°C for 45 s, followed by elongation at 72°C for 5 min. The post-PCR-generated fluorescence intensity was quantified using a QS7 System® software version. Each SNV genotyping procedure was performed in duplicate (separate experiments) for each patient. Sequenced wild-type homozygous, heterozygous, or muted homozygous patient samples were used as controls.

Radiosynthesis of ^{11}C-glyburide

^{11}C-glyburide was synthesized using a previously described automated two-step method [22]. Radiolabeling with carbon-11 was performed using a TRACERlab® FX C Pro (GE Healthcare, Buc, France). ^{11}C-CO2 was subsequently reduced to ^{11}C-CH4, iodinated to ^{11}C-CH_3I, and finally converted to ^{11}C-CH_3OTf for the first step of methylation of the precursor (5-chloro-2-hydroxy-N-(4-sulfamoylphenethyl)benzamide). The second step consisted of a reaction with cyclohexyl isocyanate heated at 70°C for 3 min. Purification was realized by reverse phase semipreparative HPLC (Waters Symmetry® C18 7.8×300mm, 7 µm) using a mixture of water/acetonitrile/TFA (45/55/0.1 v/v/v, 5 mL/min) as eluent. The purified compound was diluted with water (20 mL) and passed through a Sep-Pak® C18 cartridge (Waters, Guyancourt, France). The cartridge was rinsed with water (10 mL), eluted with ethanol (2mL) and the final compound was diluted with saline (0.9% w/v, 18 mL) before filtration under a laminar flow hood using a 0.22µm Millex® GV filter (Merck, Whitehouse Station, township de Readington, NJ, USA). ^{11}C-glyburide quality controls were compliant with the Pharmacopeia requirements. The radiochemical purity was 100% for every radiosynthesis.

Plasma analysis

The ^{11}C-glyburide fraction bound to plasma protein (f_b) with and without rifampicin infusion was estimated on the first sample (obtained before injection of ^{11}C-glyburide). The plasma sample was mixed with ~4 MBq of ^{11}C-glyburide and f_b was determined as previously described using a validated ultrafiltration method (MPS Micropartition Microcon®-YM-10 membrane, Millipore, Molsheim, France) [57].

Plasma samples obtained at 5, 10, 15, and 30 min were deproteinized with acetonitrile and injected into a UV/radioactive HPLC system. ^{11}C-glyburide was separated from its radiometabolites using a C18 semipreparative (10 × 250 mm, 10 µm) Atlantis® column (Waters, Guyancourt, France). The mobile phase consisted of 10 mM ammonium acetate in purified water (A) and acetonitrile (B). The gradient elution of B from 20% at 0 min to 90% at 12 min was applied to the column.

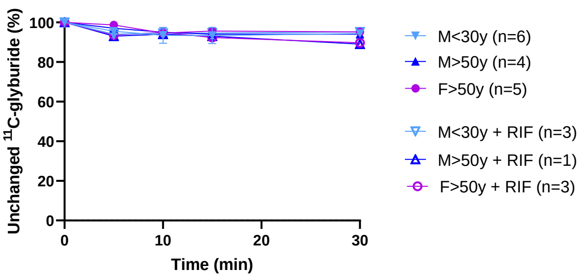

Figure S1 ^{11}C-glyburide metabolism in healthy volunteers in the absence and presence of rifampicin (RIF, 9 mg/kg i.v.). Data are mean ± SD of unchanged ^{11}C-glyburide in plasma *vs* time after i.v. injection of ^{11}C-glyburide in the baseline condition and after rifampicin infusion.

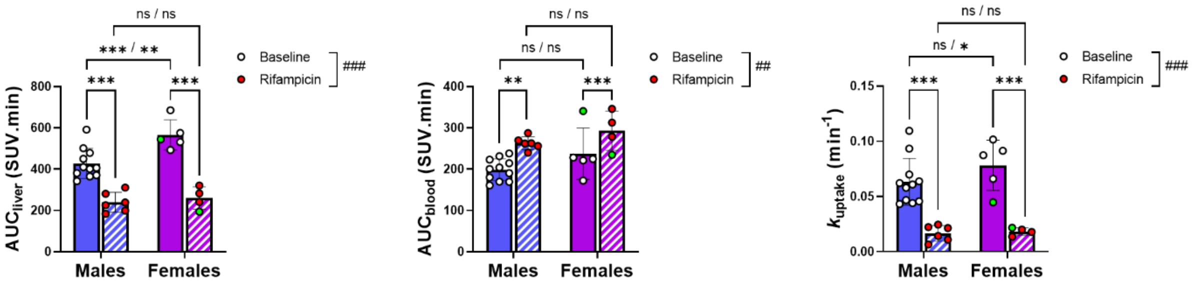

Figure S2 Areas under the time activity curves (AUC, 0-30min) in liver and blood and transfer constants (k_{uptake}) of ^{11}C-glyburide from blood to liver in males (<30y and >50y pooled) and females (>50y). k_{uptake} was assessed from integration plot analysis. White signs (and full bars) are used for baseline condition and red signs (and hatched bars) after treatment with rifampicin. The green signs indicate the subject with the homozygous mutation of the *SLCO1B1* gene. Data is mean ± SD. Statistical comparisons were performed using a mixed-effect analysis. The significance of the effect of rifampicin on the whole population is reported as $^{\#\#}P<0.01$ and $^{\#\#\#}$ $P<0.001$. The Tukey's multiple-comparison test was performed with *$P<0.05$, **$P<0.01$, ***$P<0.001$, and ns non-significant. ns/ns, ns/*, ***/** show the outcome of comparison without or with the exclusion of the green sign subject.

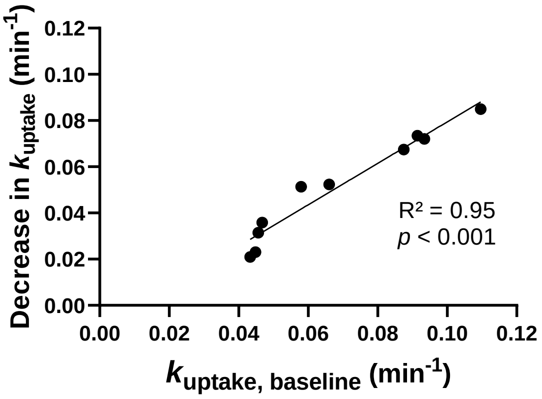

Figure S3 Correlation between the decrease in k_{uptake} for each subject after treatment with rifampicin (9 mg/kg i.v.) and the initial k_{uptake} in the baseline condition. The decrease in k_{uptake} was assessed by subtracting each subject's k_{uptake} after treatment by rifampicin from their k_{uptake} in the baseline condition. Linear regression was used to test the correlation.

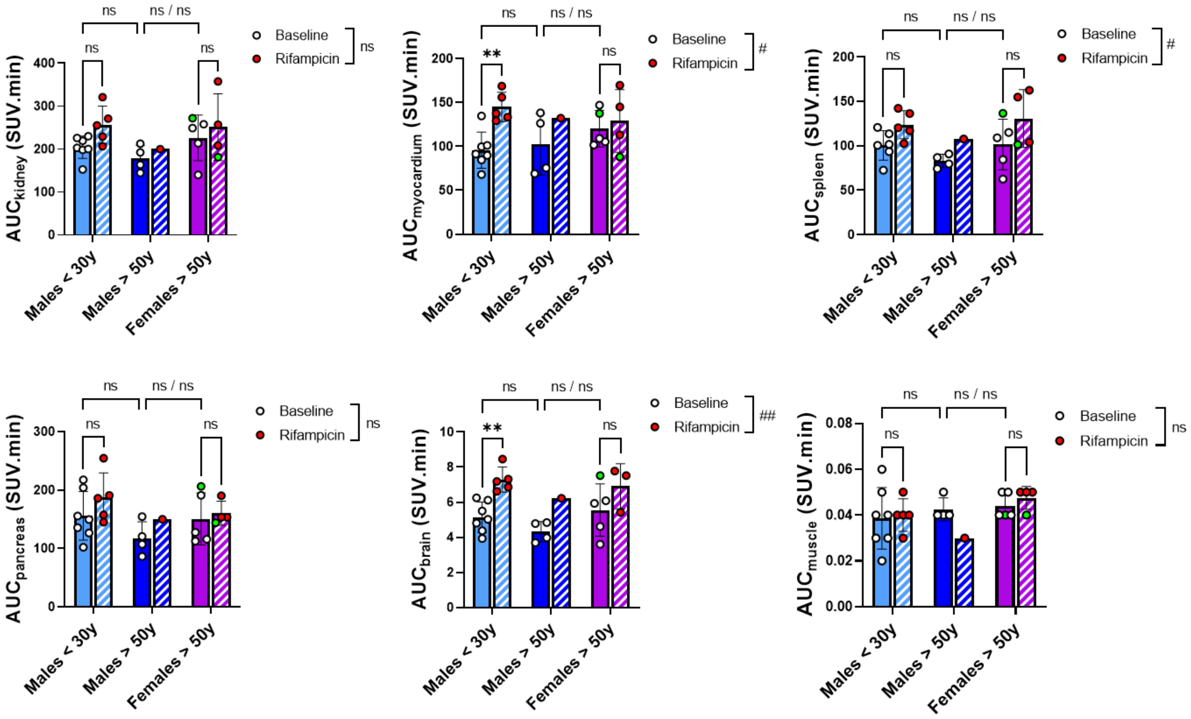

Figure S4 Areas under the time activity curves (AUC, 0-30min) of ^{11}C-glyburide. White signs (and full bars) are used for baseline condition and red signs (and hatched bars) after treatment with rifampicin. The green signs indicate the subject with the homozygous mutation of the *SLCO1B1* gene. Data is mean ± SD. Statistical comparisons were performed using a mixed-effect analysis. The significance of the effect of rifampicin on the whole population is reported as $^{\#}P<0.05$, $^{\#\#}P<0.01$, and ns non-significant. The Tukey's multiple-comparison test was performed with $^{**}P<0.01$ and ns non-significant. ns/ns shows the outcome of comparison without or with the exclusion of the green sign subject.

Table S1 Individual values of plasma protein binding, areas under the time activity curves (AUC), and ratios between AUC in each tissue and blood AUC ($K_{p,tissue}$) for ^{11}C-glyburide. AUC and $K_{p,tissue}$ were calculated from 0 to 30 min. Spleen and brain data could not be estimated in subjects 6 and 12 (rifampicin scan only) due to splenectomy and technical issues on the bed step respectively.

Group	Volunteer number	Condition	Plasma protein binding (%)	AUC (SUV.min)								$K_{p,tissue}$ (AUC_{tissue}/AUC_{blood})						
				**Blood**	**Liver**	**Kidney**	**Spleen**	**Myocardium**	**Pancreas**	**Brain**	**Muscle**	**Liver**	**Kidney**	**Spleen**	**Myocardium**	**Pancreas**	**Brain**	**Muscle**
M<30y	1	Baseline	100	169.5	410.6	203.1	72.6	89.9	102.2	4.82	9.92	2.42	1.20	0.43	0.53	0.60	0.03	0.06
	2	Baseline	99.8	238.1	501.8	230.1	120.8	99.8	204.2	3.94	5.51	2.11	0.97	0.51	0.42	0.86	0.02	0.02
		Rifampicin	100	262.3	183.5	229.4	117.0	129.3	157.7	6.86	10.30	0.70	0.87	0.45	0.49	0.60	0.03	0.04
	3	Baseline	-	231.7	381.3	219.5	93.5	101.3	155.6	5.51	6.81	1.65	0.95	0.40	0.44	0.67	0.02	0.03
		Rifampicin	99.9	270.0	199.5	255.4	102.6	133.4	186.0	7.19	9.38	0.74	0.95	0.38	0.49	0.69	0.03	0.03
	4	Baseline	98.0	170.0	478.9	198.5	100.6	68.1	126.6	4.38	8.33	2.82	1.17	0.59	0.40	0.74	0.03	0.05
		Rifampicin	97.5	263.0	281.1	271.1	142.3	137.5	190.8	8.47	12.30	1.07	1.03	0.54	0.52	0.73	0.03	0.05
	5	Baseline	99.9	204.0	406.2	200.4	101.7	91.5	149.7	6.12	6.22	1.99	0.98	0.50	0.45	0.73	0.03	0.03
		Rifampicin	99.9	256.2	238.9	206.8	136.4	168.8	145.1	7.32	9.21	0.93	0.81	0.53	0.66	0.57	0.03	0.04
	6	Baseline	99.8	161.0	342.6	152.9	-	84.7	135.5	5.03	7.13	2.13	0.95	-	0.53	0.84	0.03	0.04
	7	Baseline	99.9	223.3	592.0	226.1	113.6	134.8	217.5	6.26	9.34	2.65	1.01	0.51	0.60	0.97	0.03	0.04
		Rifampicin	99.9	287.3	311.3	321.3	120.4	156.1	254.6	6.62	11.06	1.08	1.12	0.42	0.54	0.89	0.02	0.04
M>50y	8	Baseline	99.9	180.3	371.1	163.8	87.0	75.7	120.5	4.85	7.55	2.06	0.91	0.48	0.42	0.67	0.03	0.04
	9	Baseline	99.9	200.4	365.1	144.8	79.7	69.1	107.1	3.98	8.04	1.82	0.72	0.40	0.35	0.53	0.02	0.04
		Rifampicin	99.2	240.3	225.9	200.1	107.8	132.3	150.1	6.23	7.76	0.94	0.83	0.45	0.55	0.62	0.03	0.03
	10	Baseline	100	190.6	450.5	212.9	74.3	126.4	86.5	3.69	7.74	2.36	1.12	0.39	0.66	0.45	0.02	0.04
	11	Baseline	99.9	214.2	401.8	192.6	91.0	138.2	154.5	4.79	9.96	1.88	0.90	0.42	0.65	0.72	0.02	0.05
F>50y	12	Baseline	98.9	341.0	544.6	272.2	136.5	137.6	206.3	7.52	12.16	1.60	0.80	0.40	0.40	0.60	0.02	0.04
		Rifampicin	99.9	234.2	194.0	181.5	101.4	88.2	144.2	-	8.36	0.83	0.77	0.43	0.38	0.62	0.00	0.04
	13	Baseline	99.9	228.1	685.6	258.0	109.0	147.1	127.5	5.94	10.90	3.01	1.13	0.48	0.64	0.56	0.03	0.05
		Rifampicin	99.9	346.1	320.9	357.9	154.9	169.7	190.3	7.78	15.61	0.93	1.03	0.45	0.49	0.55	0.02	0.05
	14	Baseline	99.9	224.8	493.3	213.7	115.1	105.4	191.2	4.62	8.53	2.19	0.95	0.51	0.47	0.85	0.02	0.04
		Rifampicin	97.2	312.7	282.8	257.3	162.6	145.0	153.3	7.52	15.52	0.90	0.82	0.52	0.46	0.49	0.02	0.05
	15	Baseline	99.1	221.4	575.5	249.2	84.5	109.1	112.5	6.09	10.65	2.60	1.13	0.38	0.49	0.51	0.03	0.05
		Rifampicin	99.4	278.7	241.9	208.4	104.0	113.3	153.5	5.43	14.55	0.87	0.75	0.37	0.41	0.55	0.02	0.05
	16	Baseline	99.9	172.4	532.0	140.0	62.6	101.7	115.8	3.61	7.49	3.09	0.81	0.36	0.59	0.67	0.02	0.04
